# More questions than answers: insights into potential cysteine‐rich receptor‐like kinases redox signalling in Arabidopsis

**DOI:** 10.1111/tpj.70176

**Published:** 2025-04-29

**Authors:** Sergio Martin‐Ramirez, Jente Stouthamer, Elwira Smakowska‐Luzan

**Affiliations:** ^1^ Laboratory of Biochemistry Wageningen University and Research Wageningen The Netherlands

**Keywords:** receptor redox biology, cysteine oxidation, receptor kinase, reactive oxygen species, redox proteomics, redox switch, stress response

## Abstract

Over the past few decades, significant advancements have been made in understanding how plasma‐membrane localised receptor kinases (RKs) detect signals and activate responses to various stimuli. Numerous examples of ligand‐induced receptor activation mechanisms and their downstream consequences have been characterised in detail. The crucial role of post‐translational modifications (PTMs), such as the phosphorylation of receptor kinases, has been demonstrated concerning different cellular responses. Given the diverse structures and architectures of the extracellular domains (ECDs) of RKs, it is probable that various forms of PTMs also play an essential role in receptor activation, including cysteine oxidative modifications triggered by reactive oxygen species (ROS). The function of cysteine oxidative modifications as functional redox switches that modulate protein structure and function has been extensively studied across various multicellular organisms. Based on biochemical and structural characteristics, the family of cysteine‐rich receptor‐like kinases (CRK) emerges as excellent candidates for proteins regulated in a redox‐dependent manner. This review provides a concise overview of cysteine's biochemical and structural properties in its role as a molecular redox switch. Drawing on the currently available literature, we describe how cysteine‐redox signalling is maintained, particularly in plant cells. We further focus on extracellular ROS perception and the role of CRKs as promising candidates for ROS sensors in *Arabidopsis thaliana*. We discuss the structural and biochemical properties of CRKs, their involvement in plant growth and defence processes, and our perspective on why CRKs could be key components of the ROS sensing machinery or ROS sensors, especially regarding the dimerization abilities of CRKs. Finally, we highlight the current challenges in identifying and quantifying cysteine oxidative modifications and propose methods for detecting ROS‐modified cysteines that may be promising for investigating the role of CRKs in extracellular ROS perception and signalling.

## Significance Statement

This review explores the structural and biochemical properties of cysteine‐rich receptor‐like kinases (CRKs), providing insights into their potential role as extracellular sensors of reactive oxygen species (ROS) due to the high abundance of cysteine residues in their extracellular domains (ECDs), which may undergo oxidative modifications. Furthermore, supported by literature and transcriptional databases, we summarise the significance of the CRK family in various developmental and stress response processes while underscoring the current technical and biological challenges in the field of receptor redox signalling.

## INTRODUCTION

One of the most universal plant responses to various environmental challenges is the generation of reactive oxygen species (ROS), acting as intra‐ and extra‐cellular signalling molecules (Mittler, 2017). ROS, including singlet oxygen (^1^O_2_), superoxide ($O_{1}^{∙-}$), hydroxyl radicals (OH^∙^) and hydrogen peroxide (H_2_O_2_), are essential in all living organisms as they are required for basic biological processes and stress responses (Mittler, 2017). It has been demonstrated that maintaining basal ROS levels in plants is crucial for cellular proliferation, signal transduction, differentiation and development, metabolic regulation, physiological cell death, stress acclimatisation and pathogen defence responses (Ali et al., 2018; Baxter et al., 2014; Fones & Preston, 2012; Gilroy et al., 2016; Manivannan et al., 2017; Miyakawa et al., 2007; Singh et al., 2016; Suzuki et al., 2011; Torres et al., 2006). ROS are produced in subcellular compartments, like chloroplasts, peroxisomes, mitochondria and the apoplast. These subcellular compartments also have specific scavenging machinery, ensuring that the ROS production will not exceed removal (Castro et al., 2021). Research shows that excessive production of ROS can lead to oxidative stress and cell death. Conversely, inadequate ROS production can negatively affect plant growth and defence mechanisms.

The primary processes contributing to intracellular ROS production include mitochondrial respiration, photosynthesis in chloroplasts and peroxisomal photorespiratory reactions (Mignolet‐Spruyt et al., 2016; Wrzaczek et al., 2013). On the other hand, membrane‐localised NADPH oxidases, such as respiratory burst oxidase homologues (RBOHs) and other oxidases, often activated by receptor‐like kinases (RLKs) during diverse cellular processes, are responsible for the extracellular (apoplastic) accumulation of ROS (Brandt & Hothorn, 2016; Qi et al., 2017; Wrzaczek et al., 2013). These receptors expanded enormously across the plant kingdom. For example, *Arabidopsis thaliana* (hereafter Arabidopsis) has over 600 genes that encode transmembrane RLKs and receptor‐like cytoplasmic kinases (RLCKs) (Shiu & Bleecker, 2001). RLKs perceive different ‘self’ and ‘non‐self’ derived molecules in the extracellular space and activate other RLKs and RLKCs by phosphorylation events, allowing plant cells to read signals from the local microenvironment (Belkhadir et al., 2014). RLKs, just like ROS, play critical roles in plant growth, development, immunity and symbiosis (Belkhadir & Jaillais, 2015; Couto & Zipfel, 2016; Smakowska et al., 2016). They have a tripartite structural organisation with a structurally and functionally variable extracellular domain (ECD) that perceives a broad range of signals, a single transmembrane domain (TMD), and a conserved intracellular kinase domain (KD) that transmits this signal to the inside of the cell (Santiago et al., 2016). Based on diverse ECDs, receptor kinases can be divided into 14 families, and 2 of the largest families are leucine‐rich repeat (LRR) and cysteine‐rich (CRK) receptor‐like kinases, with 225 and 44 members, respectively.

Although intracellular ROS signalling has been extensively studied and reviewed (Ali et al., 2023; Leshem & Levine, 2007; Mittler et al., 2022; Wu et al., 2023), the question of whether and how these small and highly reactive molecules are sensed and perceived in the extracellular space remains to be answered. The concept of ROS perception is based on the ability of cysteine residues to undergo reversible oxidation and reduction reactions due to cysteine nucleophilic thiol group/s, which can result in an array of oxidative post‐translational modifications (oxPTMs) (Fra et al., 2017). These reversible oxidative changes introduced on the cysteines of the protein can hypothetically serve as molecular redox switches rapidly modulating a broad range of biological processes by altering protein structure, biochemical activity, subcellular localisation and binding affinity and specificity. Such redox‐mediated regulation has been previously demonstrated for several intracellular processes, such as the regulation of various transcription factors, oxidoreductase proteins, like thioredoxins (TRXs), TRX‐like proteins and glutaredoxins (GRXs), or modulation of salicylic acid (SA) and defence signalling by NPR1 (Planas‐Riverola et al., 2021; Skryhan et al., 2015; Tada et al., 2008; Tian et al., 2018). The CRK proteins are especially interesting in the context of extracellular ROS perception, as they contain multiple cysteine residues in their extracellular domain organised in conserved (C‐X8‐C‐X2‐C) and non‐conserved motifs (Chen, 2001).

This review will first provide a concise overview of cysteine's biochemical properties and role as a molecular redox switch, specifically in plant cells. This will provide a solid background knowledge, allowing further focus on the extracellular ROS perception through cysteine residues and the role of CRKs as promising candidates for the apoplastic ROS sensors. We will discuss CRKs' structural and biochemical properties, their involvement in plant growth and defence processes, and our view on why CRKs could be components of the ROS sensing machinery. Furthermore, we will take one step forward and discuss CRK's dimerisation abilities in the context of predictive and comparative models. This feature is essential for the formation of functional protein complexes, as it represents the first crucial step that enables the transduction of perceived signals and the subsequent downstream responses, ultimately modulating specific physiological processes. Finally, we highlight challenges and bottlenecks in understanding the functional role of cysteine oxidative modifications, primarily due to challenges in detecting cysteine oxidative modifications in complex biological systems. We will thus provide an overview of methods allowing for the detection of ROS‐modified cysteine to study the role of CRKs in extracellular ROS perception and signalling.

## CYSTEINE BIOCHEMICAL PROPERTIES

Despite its very low abundance in proteins (less than 2% of all the amino acids), the cysteine residue has a prominent role in protein redox biochemistry (Bak et al., 2019; Fra et al., 2017; Leonard & Carroll, 2011). Cysteine contains a functional and highly reactive thiol group that can undergo reversible and irreversible chemical modifications in response to changing redox conditions (Garrido Ruiz et al., 2022). These modifications to the cysteine residues result from various reactive electrophilic molecules generated inside and outside the cell, like ROS, reactive nitrogen species (RNS) and reactive sulphur species (RSS) (Garrido Ruiz et al., 2022). These reactive species are produced in specific cellular organelles separated by cell membranes. Their ability to penetrate cell membranes varies based on their specific physical and chemical properties. Interestingly, O_2_, NO, and H_2_S can cross the lipid membrane by a diffusion process, while others, including H_2_O_2_ or $O_{2}^{-}$, require proteins like aquaporins or anion exchange protein channels, respectively, to facilitate transport across the membrane (Möller et al., 2019). This implies that ROS, RNS, and RSS can distally influence the redox status of the compartments where they are not produced (Baxter et al., 2014; Mittler, 2017; Qi et al., 2017). The abundance of these small molecules is highly dependent on the trade‐offs between their production and degradation pathways, and their level determines the probability of oxidation events. In that regard, the susceptibility of the cysteine thiol group to undergo oxidative modifications due to the changing redox environment becomes a crucial component of many signalling mechanisms relying on cysteine‐based redox switches (Fra et al., 2017).

Cysteine's free thiol groups can act as potent nucleophiles or reducing agents, while the corresponding disulphides can serve as electrophiles or oxidising agents (Bak et al., 2019; Garrido Ruiz et al., 2022; Paulsen & Carroll, 2013). The determinants of each cysteine thiol reactivity are given by its microenvironment within the protein (Akter et al., 2015; Bak et al., 2019; Garrido Ruiz et al., 2022). The pKa (negative base −10 logarithm of the acid dissociation constant, *K*_a_ −log_10_ *K*_a_) of the cysteine side chain, and consequently the ratio as such between the thiol (SH) and the thiolate anion (S–) state, is controlled by the local polarity, pH and interactions with neighbouring amino acid residues. If the pKa of the cysteine side chain is lower than the pH of the local environment, most of the thiols will be present as thiolates. The pKa of most thiol groups oscillates around 8–9; however, as reported previously, the pKa of protein thiol groups can range from 2.5 to up to even 12 (Bak et al., 2019).

**Figure 1:**
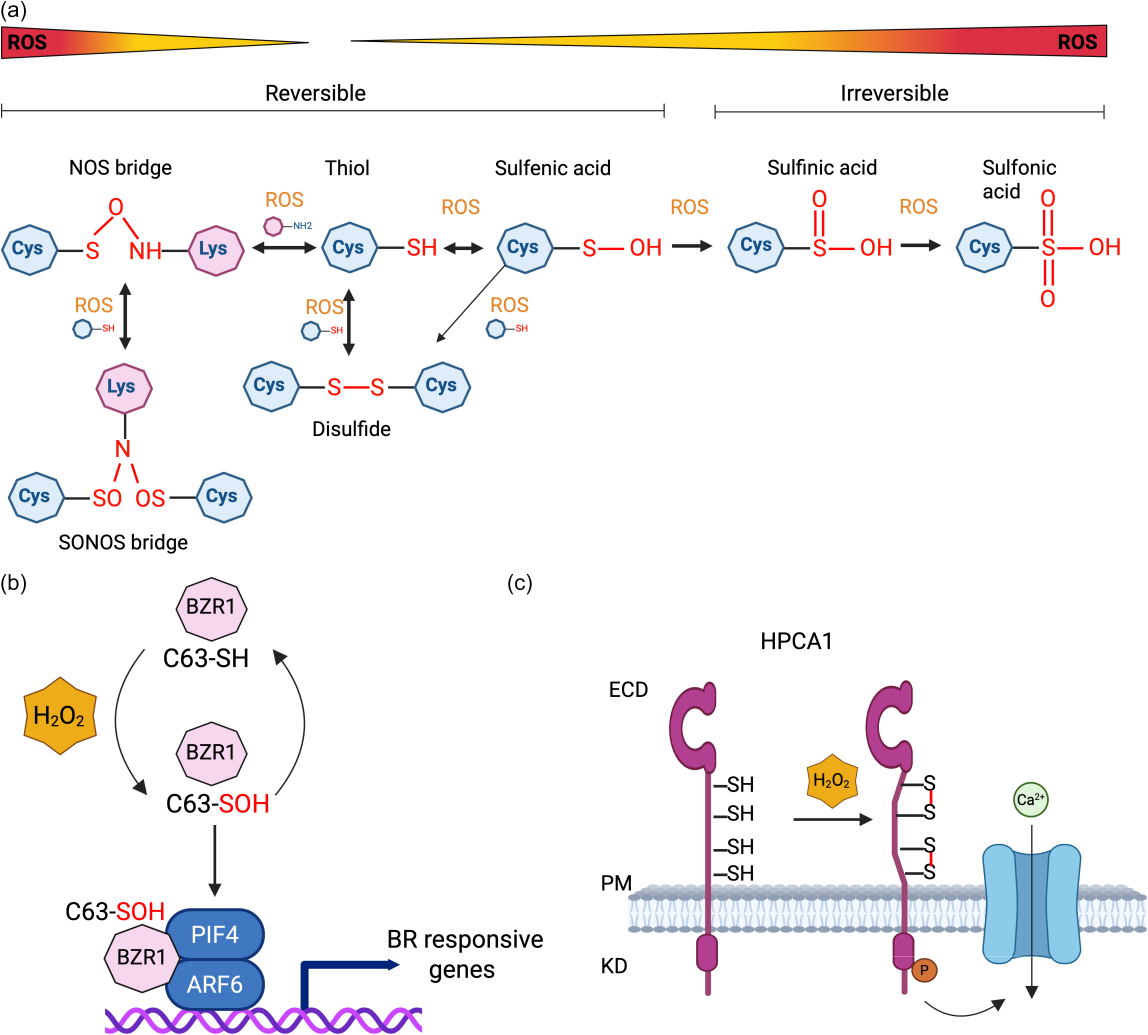
Cysteine redox modifications and their involvement in redox sensing mechanisms in plants. (a) Cysteine residues with the reactive thiol group in the presence of reactive oxygen species (ROS) can form sulphenic acid (SOH), which is also an intermediate for other modifications. Sulphenic acid can be further transformed into intramolecular or intermolecular disulphide bonds. Upon increasing the concentration of ROS, sulphenic acid can be modified into sulphinic and sulphonic acids, two types of irreversible oxidative modifications. In the left part of panel (a), there are also newly discovered NOS and SONOS bridges where cysteine is covalently linked with the lysin by the nitrogen–oxygen–sulphur bridge (NOS) or, in the case of two cysteine residues, the nitrogen atom is in a di‐oxidised (nitro) state (SONOS). (b) BRASSINAZOLE‐RESISTANT 1 (BZR1) is a transcription factor that controls brassinosteroid (BR) responses to many different abiotic stresses. Its regulation is ROS‐dependent. Upon H_2_O_2_ perception, BZR1 gets oxidised and, in that oxidised form, interacts with ARF6 and PIF4 to induce the expression of BR‐responsive genes. (c) Extracellular H_2_O_2_ can be sensed by HYDROGEN‐PEROXIDE‐INDUCED Ca^2+^ INCREASES 1 (HPCA1). HPCA1 activation is possible due to the direct oxidation of two cysteine residues. The formation of disulphide bonds leads to conformational changes that cause autophosphorylation of HCPA1 and consequently activate the Ca^2+^ channel.

In the plant cell, at the low resting pH of the apoplast (pH 5), most thiol groups are protonated and not prone to oxidation (Geilfus, 2017; Moreau et al., 2021), while at higher pH, the thiol side chain is deprotonated (S^−^), and its reactivity is greatly enhanced. Additionally, non‐enzymatic ROS scavengers, such as glutathione (GSH) and ascorbic acid (AA), are much lower in the apoplast than in the cytosol or other compartments (Lehmann et al., 2015; Podgórska et al., 2017). It has been shown that about 1–10% of the total cellular GSH is present in the apoplast, while the AA content in the apoplast has been shown to be lower by 10–100‐fold than that in the cytosol (Podgórska et al., 2017; Smirnoff, 2018). This prolongs the half‐life of H_2_O_2_, the most stable form of ROS, from milliseconds to seconds (Costa et al., 2010; Mattila et al., 2015), which is crucial for ROS accumulation, propagation and cell‐to‐cell signalling. An increasing concentration or prolonged half‐life of H_2_O_2_ results in progressive cysteine oxidation and sulphenylation (SOH). This modification is unstable and rather reactive; therefore, it can further lead to sulphinylation (SO_2_H) and sulphonylation (SO_3_H) (Figure 1a). These last two modifications are considered irreversible. Proteins with multiple cysteine residues can serve as reversible functional switches where cysteine residues, depending on the ROS levels, can exist either in their reduced state (low ROS) or oxidised state (high ROS). This can directly impact protein localization, stability and binding (Laohavisit et al., 2020; Sevilla et al., 2023; Tada et al., 2008; Tian et al., 2018; Wu et al., 2020). In the context of apoplastic redox sensors, it is crucial to consider the type of reactive molecules that may introduce chemical oxidative modifications, concentration and the amplitude of the ROS response and its duration. Recently, a new functional redox switch was discovered, where lysine and cysteine residues are covalently linked by a nitrogen–oxygen–sulphur (NOS) bridge (Figure 1a) (Rabe von Pappenheim et al., 2022; Rabe von Pappenheim & Tittmann, 2022; Wensien et al., 2021; Ye et al., 2023). The NOS bridge redox switch was first reported for the enzyme transaldolase derived from the human pathogen *Neisseria gonorrhoeae* (Wensien et al., 2021). Transaldolase enzymatic activity shifts from a largely inactive state in oxidising conditions to an active state in reducing conditions. The obtained crystal structure revealed that the redox‐active cysteine is engaged in a covalent linkage with a proximal lysine residue at the protein surface. It was demonstrated that an oxidised cysteine and a lysine residue are connected by an additional oxygen atom, forming an NOS bridge. The work of Rabe von Pappenheim et al. (2022) revealed a widespread occurrence of NOS bridges in different proteins. It was tested that of the 65 000 PDB entries, 266 deposited structures with ~400 potential NOS bridges were identified. While progress has been made in unravelling the role of cysteine oxPTMs in specific cellular and physiological processes, it has been hampered by the difficulty of detecting these modifications in complex biological systems.

## REDOX SIGNALLING IN PLANTS

In recent years, studies have shown that cysteine oxidative modifications trigger protein conformational changes, leading to changes in the activity of different kinases or transcription factors (Chi et al., 2013; Fra et al., 2017). It has become more apparent now that the presence of ROS leads to changes in specific properties of the proteins, including activity, specificity of interactions and localisation. Such changes influence the activation or suppression of stress responses and processes connected with signal transduction. A redox‐dependent functional switching from oligomers to monomers was well described for the salicylic acid (SA)‐responsive master immune coactivator, NONEXPRESSOR OF PATHOGENESIS‐RELATED GENES 1 (NPR1) (Chi et al., 2013; Tada et al., 2008). Under normal conditions without a pathogen, NPR1 remains an inactive oligomer oxidised by intermolecular disulphide bonds in the cytosol. However, during a pathogen attack, an increase in SA levels induces changes in the cellular redox state that promote the reduction of NPR1 disulphide bonds by the TRX oxidoreductases (mostly, TRX3 and TRX5) with the subsequent release of NPR1 monomers. Monomeric NPR1 translocates to the nucleus to activate pathogenesis‐related (PR) gene expression. Moreover, NO is also involved in the regulation of NPR1 oligomer‐monomer homeostasis. S‐nitrosylation of Cys156 of NPR1 promotes the formation of NPR1 oligomer, possibly by facilitating the formation of the more oxidised disulphide state at Cys156 or surrounding conserved cysteine residues. This example demonstrates a close interplay between redox‐based cysteine modifications and immune gene expression.

However, not only plant defence responses are redox‐regulated, but also processes connected with growth and development. Plants produce phytohormones Brassinosteroids (BRs) crucial for growth and stress adaptation that are perceived by membrane‐localised BRASSINOSTEROID INSENSITIVE1 (BRI1). BRI1, upon BRs perception, forms a complex with its co‐receptor BRI1‐ASSOCIATED KINASE1 (BAK1) to initiate a phosphorylation cascade activating BRASSINAZOLE‐RESISTANT1 (BZR1) (Tian et al., 2018). BRZ1 is a transcription factor regulating the expression of BR‐responsive genes. Several studies have reported that BR‐induced H_2_O_2_ accumulation is necessary for numerous BR‐mediated biological processes, including stomatal movement, salt tolerance and responses to heat and oxidative stresses. In the presence of ROS, BZR1 gets oxidised on the Cys63, and this oxidative modification enhances the interaction with AUXIN RESPONSE FACTOR6 (ARF6) and PHYTOCHROME INTERACTING FACTOR4 (PIF4). In consequence, growth‐related genes get activated (Figure 1b). The oxidised BZR1 is reduced by thioredoxin TRXh5. Various signals may influence the levels of H_2_O_2_ and TRXh5 activity; consequently, the redox regulation of BZR1 significantly contributes to the fine‐tuning of brassinosteroid responses through additional signals.

While these examples of cysteine‐rich and redox‐regulated proteins are localised within the intracellular environment, there is also evidence of redox sensing extracellularly. Plasma membrane LRR‐RKs, such as BRI1 or RECEPTOR‐LIKE KINASE 7 (RLK7), contain N‐terminal and C‐terminal caps in their ECDs, which consist frequently of two cysteine residues (Liu et al., 2013; Matsushima & Miyashita, 2012). These cysteine clusters with Cx_6_C on the N‐ and C‐terminal sides of the LRR arcs are called LRRNT (or N‐Cap) and LRRCT (C‐Cap), respectively. Both LRRNT and LRRCT form two disulphide bonds and contribute to the stability and folding, resistance to proteolytic degradation, and tertiary structure of fully matured proteins (Matsushima & Miyashita, 2012). On the other hand, another LRR‐RK, HYDROGEN‐PEROXIDE‐INDUCED Ca^2+^ INCREASE (HPCA1)/CANNOT RESPOND TO DMBQ 1 (CARD1), was recently reported to be involved in direct ROS sensing and the perception of quinones, small secondary metabolites (Laohavisit et al., 2020; Wu et al., 2020). HPCA1/CARD1 possesses a unique extracellular domain with four additional cysteines, shown by Wu et al. (2020) to be modified by extracellular H_2_O_2_ to form disulphide bonds, resulting in increased kinase activity (Figure 1c). Subsequently, active HPCA1 triggers the activation of guard cell Ca^2+^ channels and stomatal closure. Moreover, Laohavisit et al. (2020) confirmed the essential role of cysteine C395 and C405 for quinine perception, leading to the activation of downstream responses such as calcium fluxes, specific gene expression and MAPK activation. This exciting first identification of an apoplastic H_2_O_2_ sensor offers a precedent for direct chemical modification of cysteine‐containing ECDs as ROS sensors. However, HPCA1 function is limited to a particular biological context (stomatal closure), and it is therefore clear that other extracellular ROS sensors must exist. These examples demonstrate that a specific residue can act as an essential structural amino acid and does not exclude its role in the protein's function as a redox switch.

The family of cysteine‐rich receptor‐like kinases is particularly fascinating in the context of ROS signalling, as this is the sole family of RLKs characterised by such a high ratio of cysteines per extracellular domain. As it has already been extensively discussed, the presence of cysteines is the very first and likely most important determinant of the ROS sensor. Similarly to the HPCA1/CARD1 ECD cysteine residues, the oxidative modifications to the cysteine residues of CRK proteins could hypothetically function as molecular redox switches, rapidly modulating a wide array of biological processes by altering protein structure, biochemical activity, subcellular localisation and binding affinity and specificity. Additionally, as we will learn from the upcoming sections, CRKs have been primarily associated with processes involving ROS production, such as immunity, stress responses or certain developmental moments of the plant life cycle (senescence). These characteristics prompt researchers to consider them as potential extracellular ROS sensors.

## PHYLOGENETIC ANALYSIS OF CRKs

**Figure 2:**
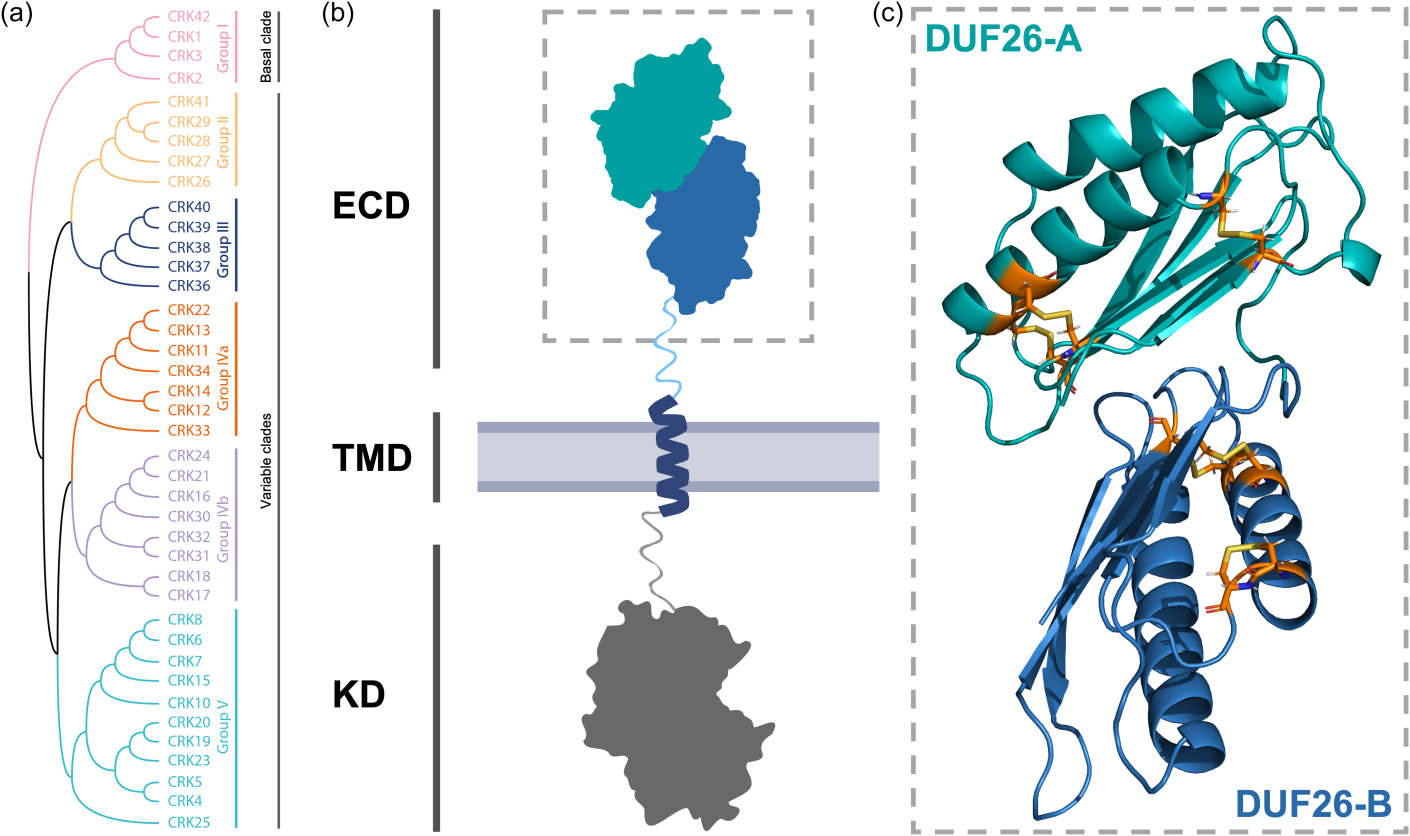
The phylogenetic relationships and structural characteristics of CRKs. (a) Phylogenetic tree of CRK family based on the extracellular domain (ECD) amino acid sequence aligned with MAFFTv7 and generated using IQtree2. The maximum‐likelihood phylogenetic tree was estimated in MEGA6 using all sites. (b) Domain structure of the CRK protein. Most typically, CKRs consist of the conserved intracellular kinase domain (KD), single‐pass transmembrane domain (TM) and variable ECD. (c) The AlphaFold model of the CRK ECD includes tandem DUF26A and DUF26B domains. The cysteines in the ECD are marked in orange.

Based on CRK ECDs' amino acid sequence alignment, CRKs can be clustered into six phylogenetic groups (Group I/II/III/IVa/IVb/V, Figure 2a) in Arabidopsis (Bourdais et al., 2015; Vaattovaara et al., 2019). According to their phylogenetic positions, CRKs are divided into two distinct clades defined as the basal CRKs clade (bCRKs) or the variable CRKs clade (vCRKs), where the basal clade is descended from a common evolutionary ancestor (paraphyletic) with respect to the variable clade (Vaattovaara et al., 2019). The basal clade (Group I) is likely older, containing sequences present in all vascular plants. CRKs that belong to this group are conserved on the sequence level, and the identification of putative orthologs from different species is frequently possible. The bCRKs evolved independently, and their extracellular domains share features that distinguish them from the vCRKs. Arabidopsis CRKs from Group I are spread out over different chromosomes. The variable clade consisting of Groups II–V is less conserved than the basal clade. All CRKs from the variable clade are located on chromosome 4, except for CRK4 on chromosome 3 (Bourdais et al., 2015; Shiu & Bleecker, 2001a, 2001b). These variable groups show several lineage‐specific expansions, originating through whole genome multiplications in angiosperms and gymnosperms, which may have led to a diversity in function (Shiu & Bleecker, 2001b; Vaattovaara et al., 2019).

## STRUCTURAL AND BIOCHEMICAL FEATURES OF CRK EXTRACELLULAR DOMAINS

CRKs have a typical receptor kinase structure consisting of an ECD, followed by a single TMD and a conserved KD (Figure 2b). The ECD is composed of a tandem repeat of domain of unknown function 26 (DUF26), designated as DUF26‐A and DUF26‐B, containing, in total, between 10 and 13 cysteine residues (Figure 2c) (Shiu & Bleecker, 2001b; Vaattovaara et al., 2019). Each DUF26 domain contains a conserved cysteine‐rich motif [Cx(8)CxxC] and additional conserved and non‐conserved cysteines distributed throughout the CRK ECD (Chen, 2001). Interestingly, DUF26 domains were also found in the CRK's closest homologues, plasmodesmata localising proteins (PDLPs), that lack KD and cysteine‐rich receptor‐like secreted proteins (CRRSPs), that contain only an ECD. PDLP5 and PDLP8 crystal structures show that each DUF26 domain consists of two α–α‐helices and five β‐strands (Vaattovaara et al., 2019). The domains are connected through hydrophobic and aromatic residues on the β‐sheets of DUF26 A and B, forming an interaction interface conserved in tandem DUF26 domains (Vaattovaara et al., 2019). Moreover, each of the 12 cysteine residues in PDLPs is shown to be involved in disulphide bridge formation (Vaattovaara et al., 2019). The number and position of cysteine residues in the extracellular domain of the bCRK (Group I) are similar to PDLPs. The remaining CRK family members have one or more additional cysteines, generally in DUF26B, that diverge from the pattern conserved in PDLPs and Group I CRKs (Figure S1). Recent advances in structure prediction with AlphaFold (Jumper et al., 2021) allowed for the modelling of CRK ECDs. The CRK ECDs show a tertiary structure reminiscent of the crystal structure of PDLP5 and PDLP8 (Figure S2a). One notable difference between CRKs and PDLPs is a differently positioned disulphide bridge in DUF26B. In PDLPs, the first β‐strand is connected to the second α‐helix through a disulphide bridge, while in CRKs, this bridge is often absent; instead, two adjacent cysteines connect to each other (Figure S2b).

**Figure 3:**
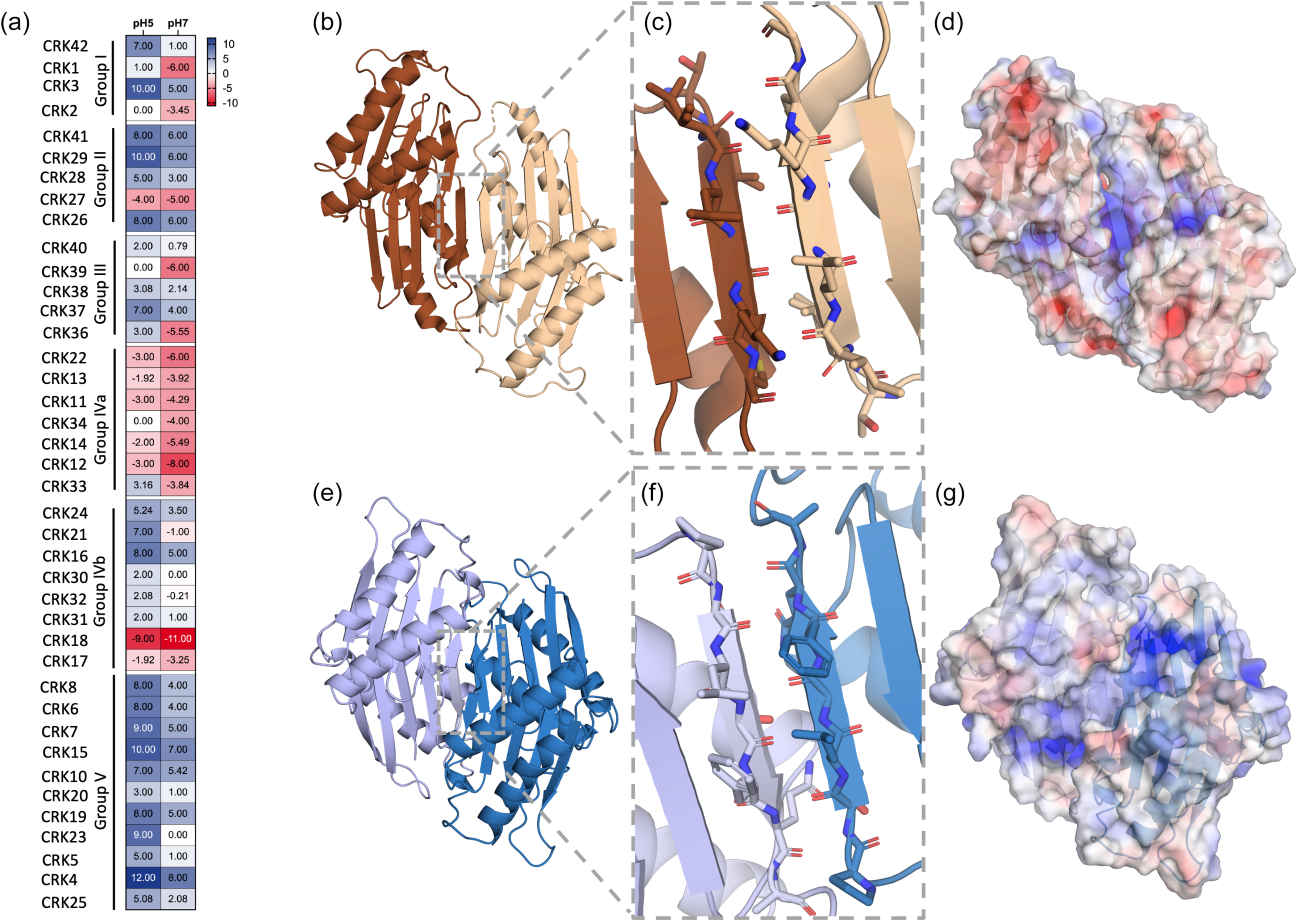
Distribution of surface charges and complex formation models of DUF26 containing extracellular domains (ECDs). (a) Distribution of surface charges at pH 5 and 7 across CRKs classified by phylogenetic affiliation. (b) The crystallographic dimer of PDLP5 (light and dark brown) (PDB:6GRE) (Vaattovaara et al., 2019). (c) The interaction interface of PDLP5 indicates that this protein forms a homodimer through the β‐strand of DUF26B, creating an extended β‐sheet. (d) Surface charge maps of the PDLP5 dimer show mildly positive charge patches near the interaction interface. Positive charges are indicated in red, and negative charges are indicated in blue. (e) AlphaFold‐Multimer predicts that CRK28 ECDs (in light and dark blue) interact similarly to PDLP5 homodimer. (f) CRK28 homodimer interaction interface. (g) The CRK28 homodimer surface charge map, similar to PLDP5 (c).

The physicochemical properties of the ECDs can be vital in modulating the strength and specificity of receptor–receptor or receptor–ligand interactions. Research has shown that size (molecular weight), hydrophobicity and topological surface charge can significantly contribute to the modulation of these interactions (Guseman et al., 2018; Requião et al., 2017; Zhou & Pang, 2018). The surface charge of the ECDs can also be necessary for the interaction between cysteine‐rich proteins (Requião et al., 2017). The unequal charge distribution at the interface of protein–protein interactions could create a molecular fingerprint where only compatible pairs of receptors interact. Amino acid charges depend on the environment, particularly on the pKa of the amino acid and on the pH of the solution (Fichman et al., 2021; Kimura et al., 2017). Apoplast alkalinization is a well‐studied process where, upon stress, the resting pH 5 of the apoplast will rapidly and transiently increase to pH 7 (Abarca et al., 2020). To provide an insight into how surface charges are distributed for the family of extracellular CRKs in Arabidopsis, AlphaFold models of all the CRK ECDs were run through the software APBS (Jurrus et al., 2018) to calculate their electrostatic potential maps at pH 5 and 7. The sum of the net surface charge of extracellular domains was calculated in Pymol, and the data is organised according to phylogenetic clustering (Figure 3a) (DeLano, 2002, 2020; Janson & Paiardini, 2021). This analysis aimed to provide insights into whether CRKs display any group specialisation based on the surface charge and if that parameter will differ between pH 5 and 7. The distribution of the surface charges shows that about 75% of Arabidopsis CRKs display positive surface charge, while the remaining 25% is generally negative. The net charge typically becomes more negative upon alkalinization, although there are some CRKs in which the change in surface charge is more pronounced. Group IVa members generally have a total negative charge at pH 5 that is further enhanced at pH 7. On the contrary, members of Group V have a high positive charge in the acidic environment that is reduced when pH rises, rendering the surface neutral or slightly positive. Additionally, Group II members are not as affected by the increase in pH. These differences in surface charge distribution can potentially influence the protein interactions of these receptors by giving specificity to each CRK for homo‐heterodimers with other signalling components. Predictions show that CRK28, which has a positive surface charge of +5, can self‐associate and create heterodimers with CRK29 (Yadeta et al., 2017), which possesses a higher positive charge of +10. The distribution of surface charge may dictate unique interaction interfaces and the accessibility of signalling components like ROS or potential ligands.

## CRK COMPLEX ASSEMBLY

RLKs canonically act as dimers, in which the ECDs facilitate receptor–receptor and receptor–ligand interactions that trigger crucial cellular responses (Han et al., 2014; Jaillais et al., 2011; Santiago et al., 2016). PDLP5 and PDLP8 crystal structures suggest a possible dimerization mechanism (Vaattovaara et al., 2019). Based on this data, the crystallographic dimers of PDLP5/8 form a continuous antiparallel β‐sheet through the first β‐strand of DUF26B (Figure 3b). AlphaFold Multimer (Evans, 2021) predicted dimer structures of CRK28 ECDs resemble the crystal structure of PDLP5 and PDLP8 (Figure 3b,e). As shown by Yadeta et al. (2017), CRK28 forms a homomeric complex on the level of full‐length receptor while transiently overexpressed in tobacco plants. The predicted CRK28‐CRK28 model (with an ipTM score of 0.79) has a similar orientation to PDLP crystallographic dimers (Figure 3d). A main interaction interface consists of hydrogen bonding of backbone amino acids in the β‐sheet surrounded by surface charge patches (Figure 3c,d,f,g), which could be important not only for dimerization with the interacting partner but also for ligand recognition and binding. However, based on the AF predictions, the amino acid composition in the β‐strand of the interaction interface is not conserved in the CRK family nor PDLPs. Such diversity in the interaction interface may allow for more versatile responses of CRKs and PDLPs. Still, whether this predicted/crystallographic interaction interface is biologically relevant remains to be assessed.

Although no ligand is known for CRKs or PDLPs, it has been demonstrated that Ginkbilobin2 (GNK2) from gymnosperm *Ginkgo biloba* seeds, which contains a single DUF26, structurally similar PDLP and CRK ECDs, can bind mannose (Miyakawa et al., 2007, 2014). This interaction was directly connected with the anti‐fungal activity of Gnk2, including the plant pathogenic Fusarium spp. Crystallography and NMR data demonstrate that Gnk2 binds mannose through N11, R93 and E104, forming an interaction interface on the β‐sheet. It is suggested that the antifungal activity of Gnk2 is driven by the recognition of mannose by hydrogen bonds. This recognition defined the carbohydrate‐binding specificity. Considering these findings, it has also been shown that in maize, upon *Ustilago maydis* infection, two DUF26‐containing proteins, AFP1 and AFP2, are transcriptionally induced and secreted to the apoplast (Ma et al., 2018). In the absence of *Ustilago*‐derived repetitive effector Rsp3 (REPETITIVE SECRETED PROTEIN 3), the AFP1/2 proteins, similarly to Gnk2, recognise and bind mannose residues and/or mannosylated proteins derived from the fungal cell wall. This recognition negatively impacts fungal cell wall integrity, resulting in fungal cell death and the release of MAMPs that induce defence responses. To prevent this, *Ustilago* secretes the effector Rsp3, which covers fungal hyphae and prevents the binding of the maize AFP proteins. The Rsp3 protein can also interact with the transmembrane DUF26 domain‐containing receptor kinases, like CRKs, to block signalling and prevent maize immune responses. Although a similar binding pocket, like that of GNK2, was found in the DUF26A domain of PDLPs, the binding of mannose or any other water‐soluble, cell wall‐derived carbohydrate has not been confirmed for tandem DUF26‐containing proteins (Vaattovaara et al., 2019). Thus, the potential ligands or interacting partners of tandem DUF26‐containing proteins, as well as the mechanism of their interactions, remain a question.

## CRK's INVOLVEMENT IN PLANT DEVELOPMENT AND RESPONSE TO ENVIRONMENTAL STRESSES

Considering the relatively high degree of amino acid sequence similarity among CRK family members, these genes were expected to be vastly redundant (Bourdais et al., 2015). However, spatial expression patterns of the CRK genes are not uniform across developmental stages and tissue/organ types (Klepikova et al., 2016). To take a closer look at this aspect, the developmental and tissue‐specific expression of the CRK family was retrieved from TravaDB and organised according to the phylogenetic groups (Figure S3) (Klepikova et al., 2016). CRKs belonging to the same phylogenetic groups tend to have similar expression patterns. Group I bCRKs are well expressed in most organs and developmental phases except for *CRK1*, specifically present only in anthers. Most Group II members, like *CRK41/29/28*/*27*, are well expressed in young tissues and roots, while Group V is primarily present in mature tissues and senescence. Certain CRKs in Groups III–V do not co‐express similarly to their clade members. Instead, their expression patterns are more similar to those of basal CRKs, such as *CRK40/17/10*, which resemble *CRK2* and *CRK3*. Interestingly, most CRKs from different phylogenetic groups are well expressed in the mature leaf or senescent tissues. CRKs' expression is differentially modulated during developmental stages but is always highly associated with developmental stages characterised by elevated ROS production (Baxter et al., 2014). Strikingly, in every phylogenetic group, at least one member, such as *CRK28* and *CRK29* (Group II), *CRK40* (Group III), *CRK11* (Group IVa), *CRK17* (Group IVb) and *CRK10* (Group V) has a relatively broad expression pattern in most organs across development. These widely expressed CRKs could function as housekeeping regulators, while others might provide calibration for secondary, more fine‐tuned responses. This mechanism would explain the intriguingly large number of CRKs in *Arabidopsis thaliana* and other species and suggests that CRKs do not function in an exclusively redundant manner (Delgado‐Cerrone et al., 2018; Hussain et al., 2022; Quezada et al., 2019).

Several studies have shown the modulation of CRK expression upon diverse abiotic stresses (Bourdais et al., 2015; Wrzaczek et al., 2010). Wrzaczek et al. (2010) showed that most family members are induced upon exposure to apoplastic ROS created by O_3_ and biotic signalling molecules related to the salicylic acid pathway. On the other hand, chloroplastic or mitochondrial generation of ROS and biotic elicitation that relies on methyl jasmonate did not trigger such a response. It has been experimentally validated that the *CRK7* gene expression, but not *CRK6*, is strongly induced upon elevated ROS treatment (Idänheimo et al., 2014). However, plants lacking CRK6 and CRK7 exhibit increased cell death after O3 exposure compared to wild type (WT) plants. Given their high sequence similarity, this may suggest partial redundancy in signalling in response to extracellular ROS. In line with these results, publicly available microarray data of abiotic treatments retrieved from eBAR (Toufighi et al., 2005) showed that CRKs form two main groups by using hierarchical clustering (Figure S4). One group is induced by osmotic stress and UV‐B light and mildly repressed by short heat shock. Osmotic, salt and heat treatments predominantly repress the second group. Similarly, as concluded by Wrzaczek et al. (2010), oxidative stress induced in the chloroplast by methyl‐viologen did not dramatically affect the expression of CRK genes. While UV‐B light, which is known to cause oxidative stress by creating ROS in the cytosol and the apoplast (Yokawa et al., 2016; Zheng et al., 2019), heavily induces the expression of the first group. Additionally, members of each phylogenetic clade distribute evenly across groups, showing no explicit clade specialisation for abiotic stress induction.

Many studies have explored the effects of single mutant T‐DNA or overexpressing lines of the CRK receptors on plant growth and development processes (Arellano‐Villagómez et al., 2021; Bourdais et al., 2015; Burdiak et al., 2015; Lee et al., 2017; Zhang, Yang, et al., 2013). However, most *crk* T‐DNA lines have a phenotype indistinguishable from WT while grown in optimal conditions, and only a few cases of mutant phenotypes have been reported. It was shown that CRK2 enhances salt tolerance at the germination stage and modulates root length. Plants lacking CRK2 (T‐DNA *crk2* mutant line) show an apparent dwarf phenotype and are more sensitive to salt stress (Bourdais et al., 2015; Kimura et al., 2020). Interestingly, the research by Li et al. (2020) showed that CRK41 has a negative regulatory effect on ABA and salt stress signalling. In relation to this matter, it has been demonstrated that CRK41 plays a vital role in the regulation of microtubule depolymerization induced by salt stress through its coordination with two MAPKs known to regulate diverse developmental and stress signalling pathways (MPK3/MPK6) (Zhou et al., 2023). Recently, it was reported that CRK4 also modulates the ABA‐signalling pathway and influences the responses to salt and drought stress in Arabidopsis. CRK5 appears to be a major player in abiotic stress signalling and growth by influencing photosynthesis (Bourdais et al., 2015; Burdiak et al., 2015). Overexpression of CRK5 leads to increased biomass and confers higher abscisic acid (ABA) sensitivity and drought tolerance (Bourdais et al., 2015; Lu et al., 2016). In plants lacking CRK5, photoinhibition was significantly enhanced, and light harvesting ability was weakened (Burdiak et al., 2015). Also, research has shown that the *crk5* mutant is characterised by enhanced senescence (Burdiak et al., 2015). The end of the reproductive phase in plants is determined by a coordinated arrest of all active meristems (also known as global proliferative arrest; GPA) in Arabidopsis, and this process was shown to be controlled by CRK14 as plants overexpressing the ECD of CRK14 demonstrate a delay of this arrest in the wild‐type background (Imai et al., 2024). Interestingly, other stress‐related responses were shown for CRK33 as plants lacking this receptor display upregulation in stomata cell fate genes, especially the expression of *TMM* and *SPCH*, suggesting a role for CRK33 in stomatal spacing in drought stress responses (Arellano‐Villagómez et al., 2021). Arabidopsis RLCK CRK45 (lacking ECD) may be involved in plant exposure to light, ultraviolet radiation, fungal elicitors, salt stress, oxidative stress, drought, salicylic acid (SA), abscisic acid and gibberellins (Zhang, Han, et al., 2013; Zhang, Yang, et al., 2013). Moreover, the work of Tanaka et al. (2012) not only demonstrated a physical interaction between CRK36 and intracellular CRK45, which are highly upregulated by abiotic stress on the transcriptional level, but also suggested that this complex formation negatively controls ABA and osmotic stress signal transduction. Growth fitness was also disturbed in CRK28 transgenic lines, as plants overexpressing this CRK have delayed flowering and are affected in silique development compared to the WT (Yadeta et al., 2017). Pelagio‐Flores et al. (2020) report the resistance phenotypes and sensitivity to ABA ability to inhibit primary root growth and seed germination in wild‐type seedlings for crk28 and 35S::CRK28 seedlings, respectively. All in all, CRK28 represents a key participant in integrating distinct environmental and developmental cues to regulate plant stress responses involving ABA signalling mechanisms at the seedling establishment stage. These reports offer a glimpse into a vastly unexplored field that will uncover the functions of CRKs in plant development processes.

## CRK's ROLE IN PLANT DEFENCE RESPONSES

To maintain proper growth, plants must constantly battle invading pathogens, which usually rely on the perception of microbe‐associated molecular patterns (MAMPs) by diverse types of RKs in the extracellular space (Belkhadir et al., 2014; Hohmann et al., 2017; Ma et al., 2016). Detecting these MAMPs by sensing receptors drives a hierarchical and stereotypic defence response that ultimately depletes microbial growth and signals to nearby uninfected cells and systemic tissues (Dangl et al., 2013; Heil & Ton, 2008). Defence responses lead frequently, but not exclusively, to ROS accumulation, ion channel exchange, defence‐related mitogen‐activated protein kinases (MAPKs) activation and transcriptional reprogramming (Chinchilla et al., 2007; Dangl et al., 2013; Ma et al., 2016).

The role of CRKs in plant defence responses has been described in the literature (Zhang et al., 2023). The induction in the transcription for the majority of CRK genes upon SA and flg22 treatments, as well as inoculation with Pseudomonas spp., was well demonstrated (Wrzaczek et al., 2010; Yadeta et al., 2017). Moreover, flg22 treatment resulted in elevated accumulation of CRK proteins (Yadeta et al., 2017). Microarray data of CRK expression was mined from the eBAR database for biotic‐challenged plants (Figure S5) (Toufighi et al., 2005). Generally, the family members fell into three broad categories: CRKs highly induced by *Pseudomonas syringae* DC3000 and *Phytophthora infestans* (*P. infestans*); CRKs that are induced by non‐host *Pseudomonas* (*P. phaseolicola*) but repressed by virulent bacteria; and CRKs that are mainly repressed by biotic stress. There is an additional group that does not cluster together with any of these groups; however, some of these CRKs are nonetheless regulated by pathogen inoculation.

CRKs have been shown to participate in the immune response by increasing resistance against bacterial pathogens (Acharya et al., 2007; Ederli et al., 2011; Lee et al., 2017; Yadeta et al., 2017; Yeh et al., 2015; Zhang, Yang, et al., 2013). High overexpression of CRKs such as CRK4, CRK5, CRK13 and CRK20 in Arabidopsis leads to cell death by HR (Acharya et al., 2007; Chen, 2001), while mild overexpression of CRK4, CRK6, CRK28, CRK36 and CRK45 confers higher resistance to *Pseudomonas* DC3000 (Lee et al., 2017; Yadeta et al., 2017; Yeh et al., 2015; Zhang, Yang, et al., 2013). Regardless of the expression level, the increased immunity output of these CRKs is related to salicylic acid and involves increased levels of pathogenesis‐related proteins such as PR1. CRKs have been found to boost immunity by affecting ROS burst; therefore, they are signalling components upstream of RBOHs (Castro et al., 2021; Kimura et al., 2017; Lee et al., 2017). In fact, CRK2 directly interacts with RBOHD and phosphorylates its C‐terminal end at S703 upon flg22, which leads to an increase in the oxidase activity of RBOHD (Bourdais et al., 2015; Kimura et al., 2020). Additionally, CRK5 and CRK22 were shown to regulate the defence responses against *Verticillium dahliae* toxins (Vd‐toxins) in Arabidopsis (Zhao et al., 2022). Recently, it was revealed that the gain‐of‐function allele of CRK10 causes the collapse of xylem vessels and enhances the immune response in Arabidopsis (Piovesana et al., 2023). Also, CRK36 was shown to regulate flg22‐triggered immunity responses, likely through in vivo association with FLS2 and BIK1. interestingly, CRK36 enhanced the phosphorylation of BIK1 triggered by flg22, which exhibited deficiencies due to cysteine mutations in the DUF26 motifs of CRK36 (Lee et al., 2017). Taken collectively, these findings indicate that CRKs contribute to both the primary and secondary signalling pathways of PTI, potentially enhancing the perception of ROS in a local and/or systemic manner.

## CHALLENGES AND PERSPECTIVES IN RECEPTOR REDOX BIOLOGY

While the importance of CRKs in developmental, environmental and defence response is evident, little is known about the precise molecular mechanisms of CRK activation and signal transduction. Most published data report modifications to the plant phenotype of different transgenic lines, which (with the exception of the knockout line of CRK2, basal clade) are quite subtle and likely depend on gene dosage, particularly for lines overexpressing certain CRKs. It has been proposed that CRK genes display a considerable degree of redundancy, based on phylogenetic studies and the distribution of variable clades in the tandem repeats on chromosome 4 (Bourdais et al., 2015; Shiu & Bleecker, 2001a, 2001b). Therefore, the most logical approach would be to begin examining the higher order mutants for the closest phylogenetic homologues. However, this was impossible before the era of CRISPR‐Cas due to the previously mentioned rather peculiar arrangement of most Arabidopsis vCRKs on a single chromosome. Secondly, studying specific cellular and physiological responses without knowing which molecule activates the CRK(s) of interest poses another challenge. This primarily hinders research on the activation mechanisms and downstream responses that have actual consequences for modulating plant growth and stress responses. Therefore, whether this is the canonical type of signalling molecule, such as a peptide, glycan, or hormone, or a highly reactive molecule like ROS through cysteine modifications, identifying the mechanisms of CRK activation is the essential and fundamental step towards a deeper mechanistic understanding of complex formation abilities and signalling processes.

The long‐postulated, yet thus far not experimentally proven, role of the CRK family is extracellular ROS sensing. Oxidative cysteine modifications play a fundamental role in redox signalling, regulation of proper protein function, and, ultimately, maintaining cellular processes. The biological significance of these chemical modifications has been increasingly demonstrated in recent years. However, unravelling the role of cysteine oxidative modifications for specific cellular and physiological functions has been hampered by the difficulty in detecting these modifications in complex biological systems. This is even more complex when it comes to mapping these modifications on the plasma membrane‐localised proteins (like CRKs) due to the difficulties of the protein extraction approaches, protein abundance, and hydrophobicity. A broad spectrum of direct or indirect approaches was developed to identify reversible or irreversible cysteine oxidative modifications using gel‐based or mass spectrometry‐based approaches (Artemenko et al., 2015; Duan et al., 2017; Lennicke et al., 2016; Leonard & Carroll, 2011; Parker et al., 2015; Pham et al., 2021; Van Der Reest et al., 2018). Irreversible modifications (sulphinic/sulphonic acids) typically have good stability under sample preparation and liquid chromatography–mass spectrometry (LC–MS) thus, direct identification of these modifications is entirely feasible but requires sensitive equipment. On the contrary, identifying reversible modifications in complex biological samples is challenging because of the labile nature and low abundance of these modifications. Additionally, artificial oxidations can be introduced during sample preparation and conversion between different forms of reversible PTMs during sample processing. Therefore, efficient enrichment approaches are required to reduce sample complexity and enhance detection levels, especially for measuring endogenous levels of redox PTMs.

**Figure 4:**
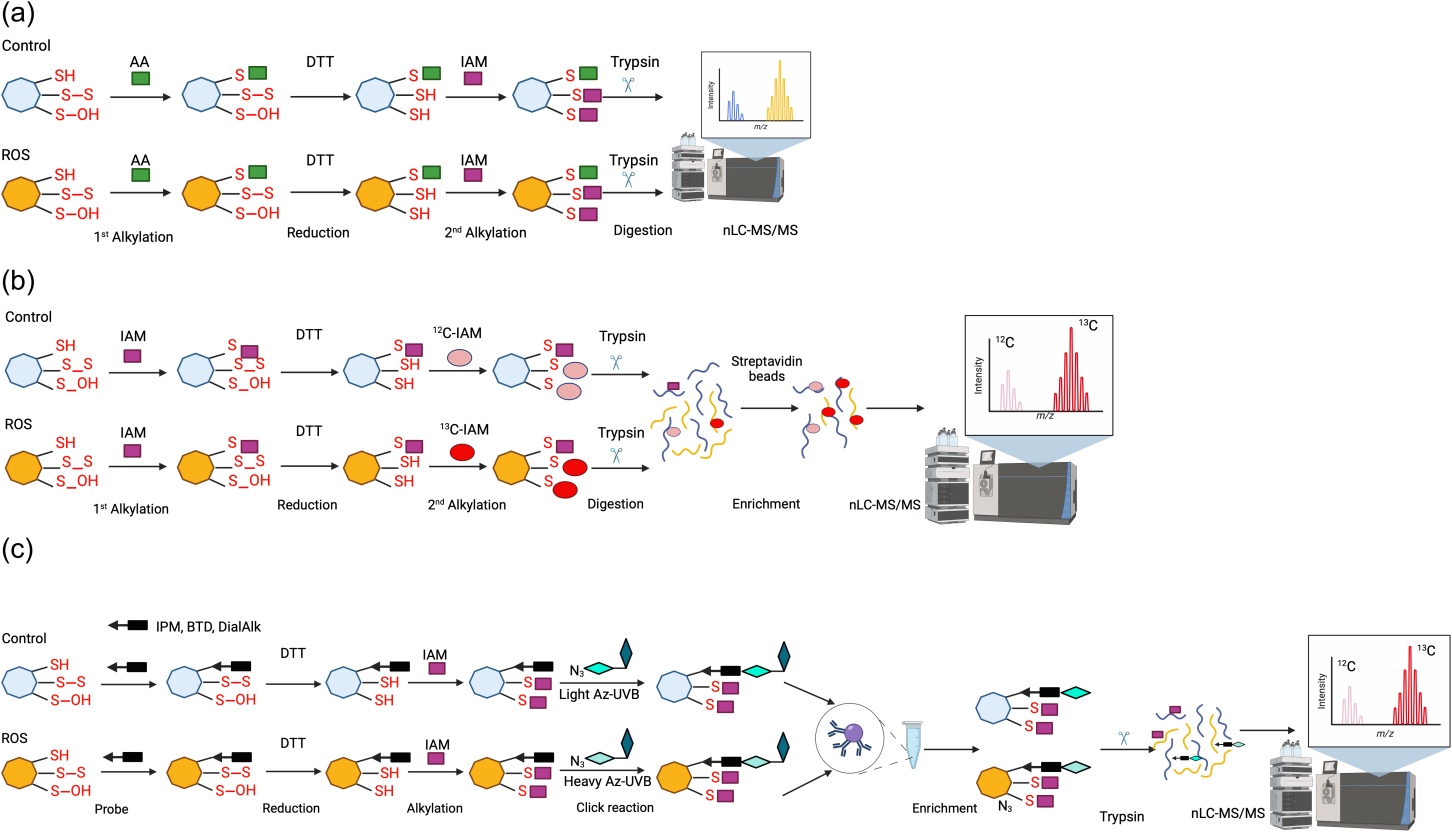
An overview of proteomics‐based approaches for the quantitative identification spectrum of cysteine oxidative modifications. (a) The differential alkylation method (DAM) is a technique that labels reduced thiols using an alkylation agent, such as acrylamide (AA) or iodoacetamide (IAA). This is followed by the reduction (DTT) of all reversibly oxidised sites and a second alkylation with another alkylating agent, such as iodoacetamide (IAM) or *N*‐ethylmaleimide (NEM). This approach facilitates the identification and quantification of oxidative modifications to cysteine. (b) The isotope‐coded affinity tag (ICAT) is a method that uses biotinylated IAM with isotopically light and heavy linkers. Cysteines containing free thiol groups are first labelled with biotinylated light IAM. Sequentially, oxidised cysteines are reduced with DTT and labelled with biotinylated IAM with a heavy isotope. Afterwards, biotinylated‐IAM proteins are enriched on the streptavidin beads, digested with trypsin, and analysed by quantitative MS. This method provides a high‐resolution view of cysteine reactivity changes. (c) Isotopic tandem orthogonal proteolysis‐activity‐based protein profiling (isoTOP‐ABPP) exemplifies a click‐chemistry strategy. IA‐alkyne probes target proteins featuring free thiol groups across two different proteomes. Next, following DDT reduction, IAM labels remaining protein‐free thiol groups. To link isotopically labelled cleavable biotin‐azide tags (L and H) with IA‐alkyne labelled proteomes, copper(I)‐catalysed azide‐alkyne cycloaddition (CuAAC) is utilised. The doubly labelled proteomes are mixed, followed by affinity purification using streptavidin beads. Following purification, on‐bead tryptic digestion and mass spectrometry identification represent the final steps in this technique. Calculating the heavy/light ratio reveals changes in cysteine reactivity between the two proteomes for each cysteine‐containing peptide that is labelled. This method enables high‐throughput identification and quantification of cysteine oxidative modifications.

Using proteomics approaches to map cysteine oxidative modifications is critical for fundamental redox biology (McConnell et al., 2019). MS has outstanding potential in systematic and large‐scale assessment of the presence and quantity of oxidative modifications. The broad impact of ROS on plant proteomes was collectively highlighted in several scientific publications demonstrating proteome‐wide mining of ROS‐sensitive cysteines in various plant species (Alvarez et al., 2011; Liu et al., 2014; Slade et al., 2015; Wang et al., 2012). Significant challenges with profiling such oxidative modifications are still connected to cellular contexts and quantification of absolute levels of such PTMs. Currently, different strategies are used to study the dynamic reversible oxidation of protein cysteines (Pham et al., 2021) (Table S1). There are methods based on the principles of the differential alkylation method (DAM), wherein reduced thiols are blocked by an alkylation agent, such as acrylamide (AA), followed by reduction of all reversibly oxidised sites, and second alkylation with another alkylation agent, like iodoacetamide (IAM) or NEM (Figure 4a) (Anjo et al., 2019; Wang & Kaltashov, 2015; Wojdyla & Rogowska‐Wrzesinska, 2015). This simple method doesn't require any enrichment strategies and uses costless chemistry. However, partly due to the lack of enrichment, this method is less suitable for lowly abundant proteins and proteins with relatively low oxidation stoichiometry. Furthermore, the DAM approach doesn't allow for a specific distinction between the formation of disulphide bridges and sulphenylation. A similar approach was used to identify cysteine oxidative modification on the HPCA1 receptor protein. Especially in a complex biological sample, enrichment methods, like isotope‐coded affinity tags (ICAT) or iodoacetyl isobaric tandem mass tags (IodoTMT), are broadly used (García‐Santamarina et al., 2014; Lennicke et al., 2016; McConnell et al., 2019; Pham et al., 2021; Van Der Reest et al., 2018). The ICAT uses biotinylated IAM containing isotopically labelled light and heavy linkers (Figure 4b). Cysteines containing free thiol groups are first labelled with biotinylated light IAM. Sequentially, oxidised cysteines are reduced with the appropriate reducing agent and labelled with biotinylated IAM with a heavy isotope. Afterwards, biotinylated‐IAM proteins are enriched on the streptavidin beads, digested with trypsin, and analysed by quantitative MS. The IodoTMT works based on the same principles, but on the peptide levels. The advantage of using isobaric tags is that they allow for multiplexing, meaning analysing multiple samples in one experimental approach (Afiuni‐Zadeh et al., 2016; Prakash et al., 2018; Van Der Reest et al., 2018). Those methods ultimately offer better coverage of the cysteine‐containing peptides but are usually more challenging from the technical and data processing point of view. The newly emerging trend is click chemistry (orthogonal chemistry)‐based approaches, where different probes can be used against various oxidative modifications, followed by a reaction with isotope‐labelled biotin tags (Pham et al., 2021) (Figure 4c). Generally, click chemistry‐based approaches are based on three essential steps: alkylating reduced cysteines in proteins with a probe, click chemistry‐based incorporation of isotopically labelled cleavable tags, protein purification and on‐bead digestion (Harris et al., 2023; Parker & Pratt, 2020). Typically, the probes consist of one group that reacts with reduced cysteines and the second one, an alkyne group, used for tagging with the Heavy (H) and Light (L) TEV‐biotin tag click chemistry. These tags consist of three modules: a biotin group for streptavidin affinity purification, a TEV protease recognition site fused with an isotopically labelled Valine, and a so‐called azide handle for click chemistry. IA‐alkyne probes label proteins with free thiol groups from two different proteomes in an approach like isoTOP‐ABPP (isotopic tandem orthogonal proteolysis‐activity‐based protein profiling). in the next step, Copper(I)‐catalysed azide‐alkyne cycloaddition (CuAAC) is employed to connect isotopically labelled cleavable biotin‐azide tags (L and H) with IA‐alkyne‐labelled proteomes. Doubly labelled (with H and L isotopes) proteomes are combined, and streptavidin beads for affinity purification are performed. After purification, on‐bead tryptic digestion and MS identification are portrayed as the last steps of this approach. A calculated heavy/light ratio will show cysteine reactivity changes between two proteomes for each labelled cysteine‐containing peptide. There is an increasing variety of chemoselective probes available targeting different types of oxidative modifications, like IPM is an iodoacetamide‐based alkyne probe for cysteinyl thiol (–SH), BTD is a benzothiazine‐based alkyne probe for cysteine sulphenic acid (–SOH), and DiaAlk is a diazene‐based alkyne probe for cysteine sulphinic acid (–SO_2_H) (Meng et al., 2021). The BDT probe was successfully applied to the Arabidopsis cell culture to reveal the S‐sulphenylation landscape at the cysteine site level (Huang et al., 2019).

Each method presented in this paragraph enables the quantitative and site‐specific mapping of redox‐mediated changes to protein amino acids (Table S1). However, it is essential to realise that aside from such exploratory screens, additional validation of the oxidised cysteines discovered is required. Given the ongoing advances in this field, fascinating times await plant redox research. These will all be very helpful for a better understanding of the roles of various classes of proteins, including CRKs, in ROS perception and signalling to maintain redox‐driven cellular responses.

## CONCLUSION

**Figure 5:**
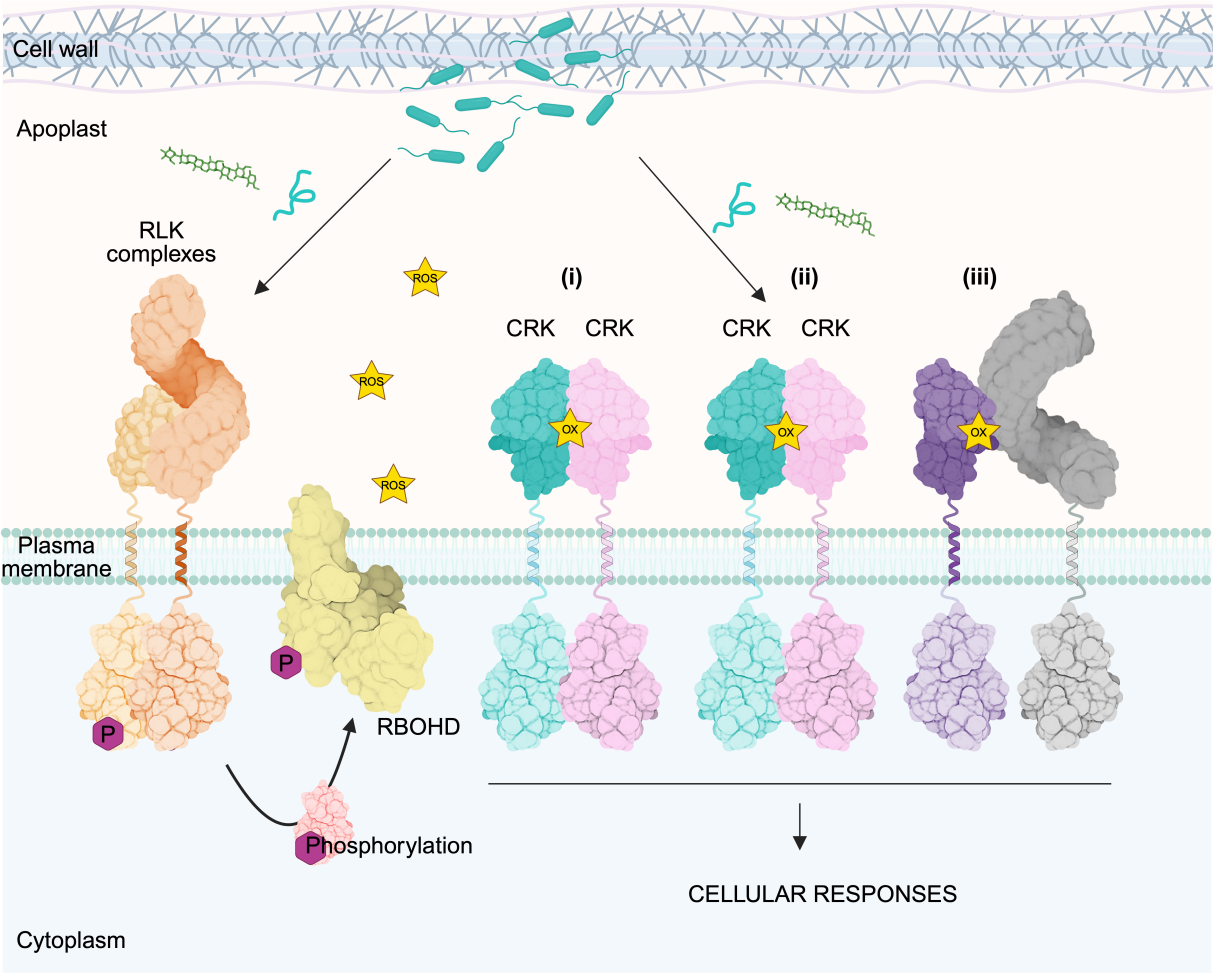
Proposed mechanism of the reactive oxygen species (ROS) sensing by CRKs. In the presence of the ligands (for example, peptides derived from pathogen or glycans derived from pathogen and/or plant cell wall), there is an activation and a very rapid complex formation between ligand‐sensing RLK and its co‐receptor. A good example here is an activation and a complex formation between FLAGELLIN SENSITIVE 2 (FLS2) and BRI1‐ASSOCIATED KINASE 1 (BAK1) upon sensing of flagellin 22 (flg22), a 22‐amino acid epitope derived from bacterial flagella. This leads to the activation and phosphorylation of substrates, such as RBOHs, resulting in ROS burst and alkalization of the apoplast. Increasing the extracellular pH enables cysteine oxidative modification of CRKs and modulation of their interactions to stimulate downstream signalling and cellular responses (i). Also, as mentioned before, ligands in the form of peptides or glycans, which are secreted into the apoplast under diverse stimuli, can play a role in the modulation of ROS‐triggered CRK interactions either with other CRK members (ii) and/or members of other RLKs families (iii) leading to activation of downstream cellular responses.

This work offers a thorough overview of cysteine biochemistry and several established mechanisms of redox regulation. We summarised the current knowledge about the RLK family of CRKs, which, based on biochemical and structural characteristics, emerge as potential yet unproven candidates for ROS sensing proteins. We provided further insight into this family of proteins by integrating bioinformatically determined physicochemical properties of the modelled CRK ECDs and analysing the expression patterns throughout the development and stress induction of the Arabidopsis CRK family. Considering the relatively limited amount of available literature, the precise role of CRKs in redox‐dependent developmental and stress signalling remains to be discovered. Although still largely speculative, it may be that when a critical concentration of ROS in the apoplast is reached – triggered by various defence and development‐related processes – certain CRKs could become more susceptible to oxidative cysteine modifications (Figure 5). Depending on the biochemical and structural properties, such as surface charge or accessibility of cysteine residues, CRK ECDs would exhibit differential susceptibility to oxidative modifications. Oxidation of cysteine residues in CRK ECDs could lead to differential CRK complex formation, phosphorylation events, and subsequent signal transduction. These modifications are likely to introduce slight structural rearrangements of the CRK ECDs, facilitating specific interactions with other receptor kinases or dissociation from preformed complexes, thereby acting as positive or negative regulators of various signalling pathways. Another possibility to consider is CRK activation upon ligand binding. This still somewhat speculative model of ROS perception fundamentally differs from the canonical reversible receptor‐ligand interactions, as ROS would not remain bound but would instead chemically modify the receptor or sensor. Although this theory has never been experimentally explored, it cannot be ruled out that CRKs perceive, for instance, ligands in the form of peptides or glycans in enhanced ROS production. Given that these scenarios require thorough experimental testing and validation, the importance of employing specific, sensitive and reliable methodologies to investigate the dynamics of oxidative modification becomes a critical focus within the domain of plant redox biology (Boxes 1 and 2).

Box 1 **Bullet point summary**

1. ROS are important signalling molecules in plants, essential for processes such as cellular proliferation, signal transduction, development, and pathogen response defence.
2. Cysteine residues can act as molecular redox switches, modulating various biological processes.
3. The redox status of cysteine influences protein structure, activity, and localization.
4. CRKs are candidates for sensing extracellular ROS due to their abundance of cysteine residues.
5. CRK genes show variable expression across developmental stages and stress responses, indicating specialised functions.
6. Detecting oxidative modifications of cysteine in complex biological samples remains challenging due to the labile nature of reversibly modified cysteines.

Box 2 **Open questions**

1. How do specific cysteine residues in CRK ECDs facilitate the perception and signalling of ROS in plants?
2. Given the phylogenetic diversity of CRKs, what are the distinct functional roles of each group of CRKs in regulating plant responses to abiotic and biotic stresses?
3. What are the precise signalling pathways and mechanisms through which oxidative modifications of cysteine residues in CRKs influence their structural dynamics and functional interactions?
4. How might ligands, if any, interact with CRKs in the context of ROS signalling, and how does this interaction differ from conventional receptor‐ligand binding mechanisms?
5. How might the current proteomics approaches for detecting and quantifying oxidative modifications of cysteine in complex biological systems be improved to enhance our understanding of redox signalling in plants?

## AUTHOR CONTRIBUTIONS

SM‐R, JS, and ES‐L wrote the manuscript.

## CONFLICT OF INTEREST

The authors declare no conflicts of interest.

## Supporting information


**Figure S1.** Sequence alignment of PDLP5 and one representative of each phylogenetic group of the CRK family.
**Figure S2.** (a) The crystal structure of PDLP5 (Beige, PDB:6GRE) aligned with the AlphaFold model of the CRK28‐ECD (Blue). (b) Disulfide bridge in PDLP5 (C148–C215) that is not conserved in variable clade CRKs.
**Figure S3.** CRK expression patterns across various organs and developmental stages.
**Figure S4.** Cluster analysis of *CRK* expression in response to abiotic stress.
**Figure S5.** Cluster analysis of *CRKs* expression in response to biotic stress.

**Table S1.** Overview of redox proteomics methods for identifying oxidative modifications of cysteine.

## Data Availability

Data sharing is not applicable to this article as no new data were created or analyzed in this study.
