# Supplementary material for: More questions than answers: insights into potential cysteine‐rich receptor‐like kinases redox signalling in Arabidopsis
Source: Plant J. 2025 Apr 29;122(2):e70176. doi: 10.1111/tpj.70176 (PMC12040379; doi:10.1111/tpj.70176)
Supplement: Supplementary file 1 — Figure S1. Sequence alignment of PDLP5 and one representative of each phylogenetic group of the CRK family. Figure S2. (a) The crystal structure of PDLP5 (Beige, PDB:6GRE) aligned with the AlphaFold model of the CRK28‐ECD (Blue). (b) Disulfide bridge in PDLP5 (C148–C215) that is not conserved in variable clade CRKs. Figure S3. CRK expression patterns across various organs and developmental stages. Figure S4. Cluster analysis of CRK expression in response to abiotic stress. Figure S5. Cluster analysis of CRKs expression in response to biotic stress. [file TPJ-122-0-s002.docx]

**More Questions than Answers: Insights into Potential Cysteine-Rich Receptor-Like Kinases Redox Signalling in Arabidopsis**

Sergio Martin-Ramirez^1^, Jente Stouthamer^1^, Elwira Smakowska-Luzan^1^

^1^ Wageningen University & Research Laboratory of Biochemistry, Wageningen, The Netherlands

Supplementary data figures:

Supplementary Figure 1

Supplementary Figure 2

Supplementary Figure 3

Supplementary Figure 4


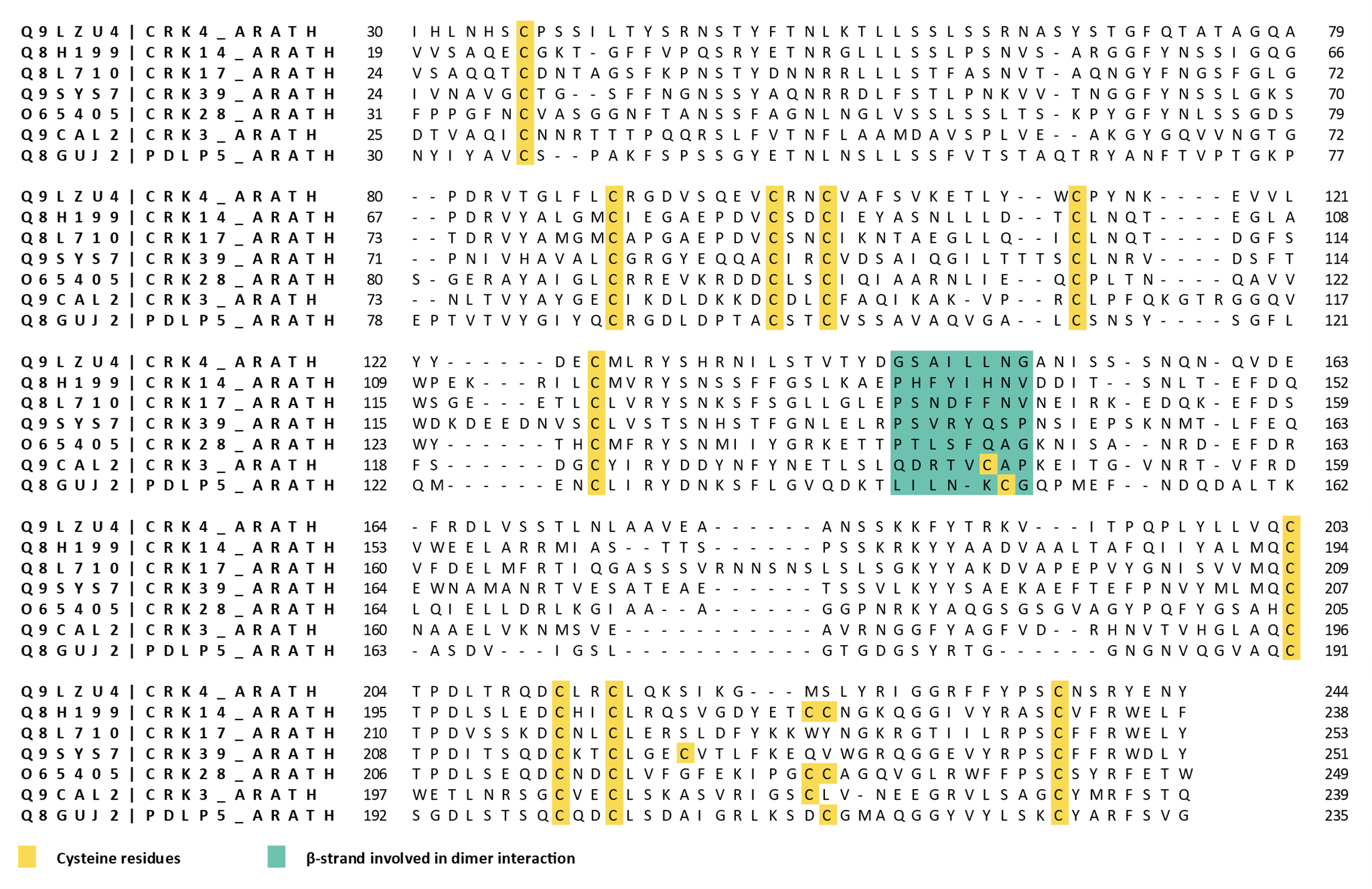


**Supplementary Figure 1. Sequence alignment of PDLP5 and one representative of each phylogenetic group of the CRK family.** Cysteine residues are highlighted in yellow, β strand involved in dimer interaction in (sequence alignment performed with T-coffee)


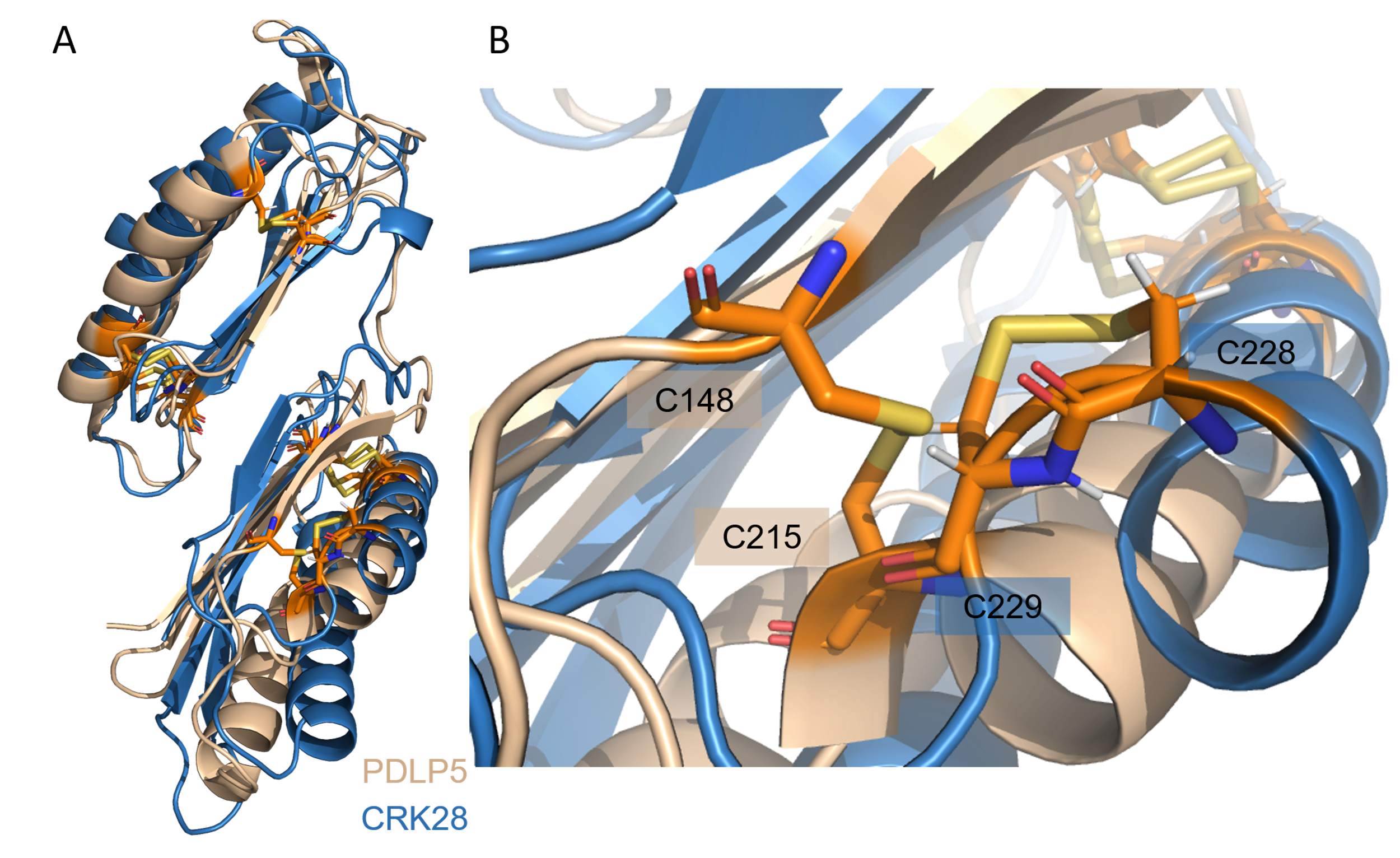


**Supplementary Figure 2.** A) The crystal structure of PDLP5 (Beige, PDB:6GRE) aligned with the AlphaFold model of the CRK28-ECD (Blue). B) Disulfide bridge in PDLP5 (C148-C215) that is not conserved in variable clade CRKs. CRK28, shown here, has a predicted disulfide bridge connecting two sequence adjacent cysteines (C228-C229) instead.


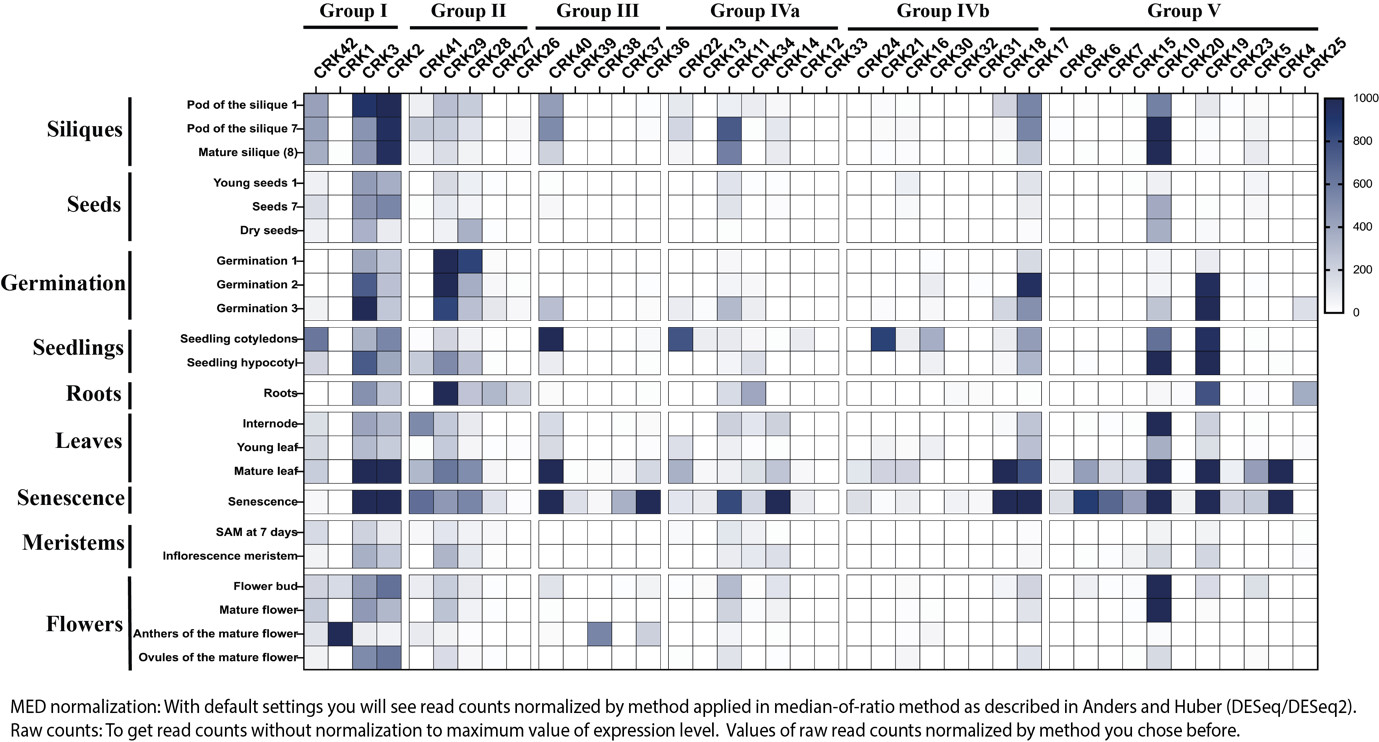
**Supplementary Figure 3. CRK expression patterns across various organs and developmental stages.** Data mined from Klepikova atlas (TravaDB). CRK family members are organized based on phylogeny. The scale represents absolute expression values from 0 to 1000 counts. MED normalization was applied. With the default settings, the read counts were normalized by the method applied in the median-of-ration method as described by Anders and Huber (DESeq/DESeq2). Values over 1000 are colour-saturated.


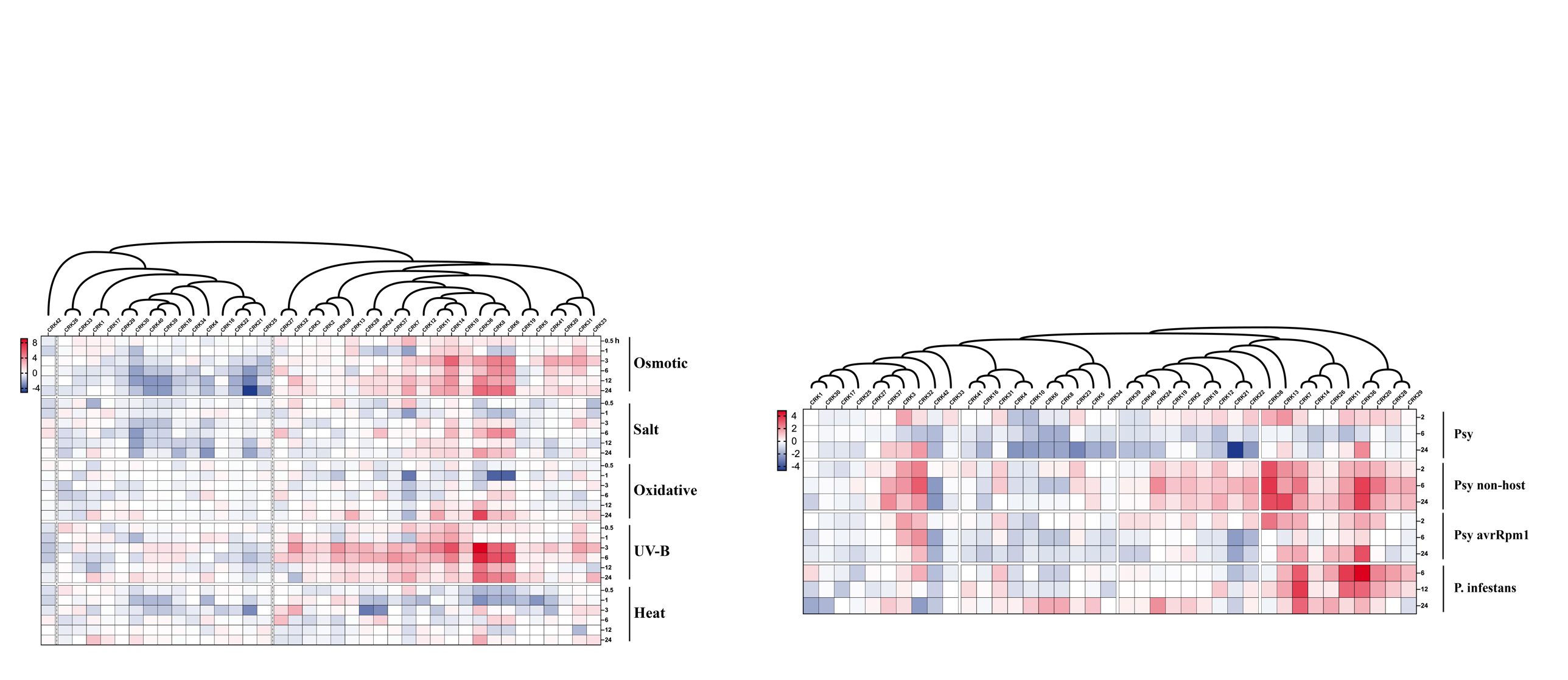


**Supplementary Figure 4. Cluster analysis of *CRK* expression in response to abiotic stress.** Publicly available *Arabidopsis* microarray data was clustered to reveal patterns in the transcriptional regulation of *CRKs* using Hierarchical Clustering. Blue and red indicate increased or decreased expression compared to untreated plants, respectively. The intensity of the colors is proportional to the absolute value of the fold difference.


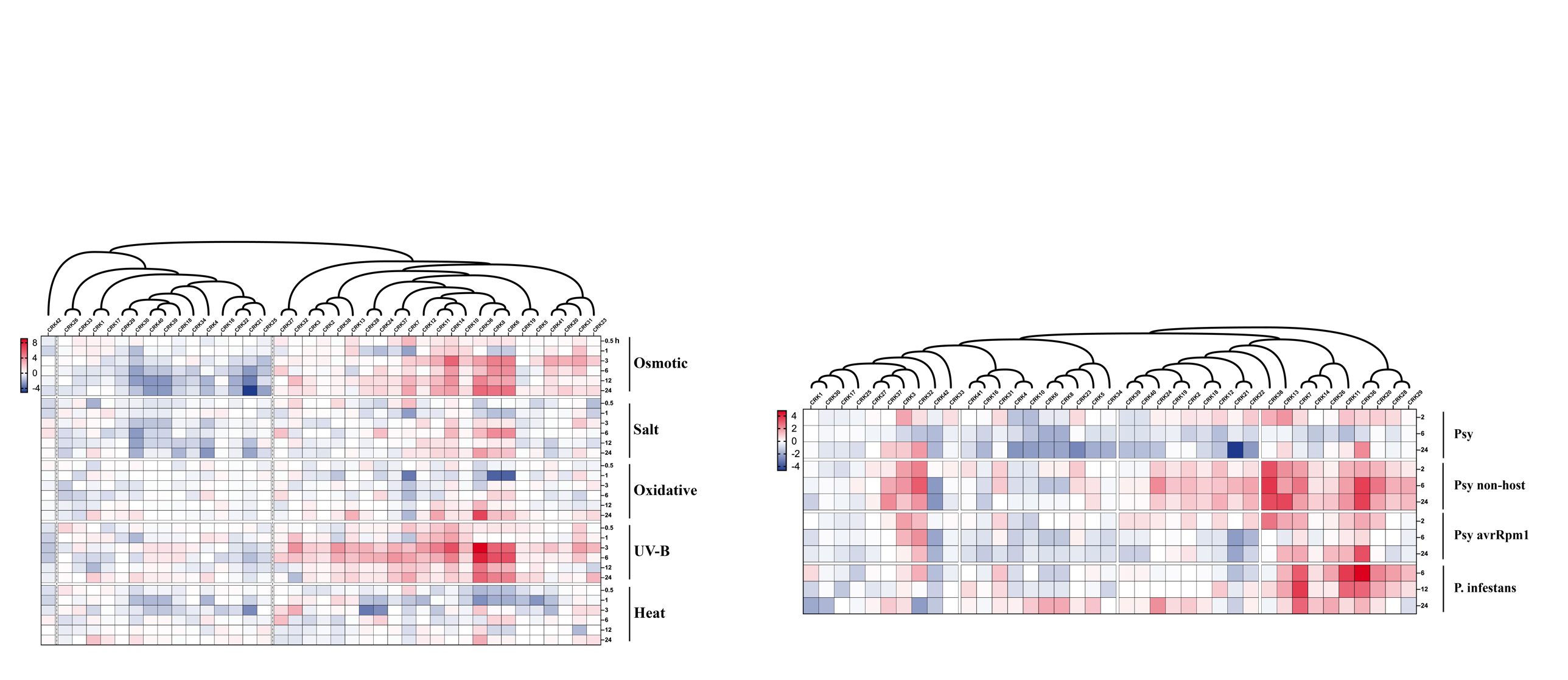


**Supplementary Figure 5. Cluster analysis of *CRKs* expression in response to biotic stress.** Publicly available *Arabidopsis* microarray data was clustered to reveal patterns in the transcriptional regulation of *CRKs* using Hierarchical Clustering. Blue and red indicate increased or decreased expression compared to untreated plants, respectively. The intensity of the colours is proportional to the absolute value of the fold difference.
